# High expression of RNF169 is associated with poor prognosis in pancreatic adenocarcinoma by regulating tumour immune infiltration

**DOI:** 10.3389/fgene.2022.1022626

**Published:** 2023-01-05

**Authors:** Jieyan Wang, Hanghang Chen, Qiong Deng, Yeda Chen, Zhu Wang, Zhengzheng Yan, Yinglin Wang, Haoxuan Tang, Hui Liang, Yong Jiang

**Affiliations:** ^1^ Department of Urology, The People’s Hospital of Longhua, The Affiliated Hospital of Southern Medical University, Shenzhen, Guangdong, China; ^2^ Guangdong Provincial Key Laboratory of Proteomics, State Key Laboratory of Organ Failure Research, Department of pathophysiology, School of Basic Medical Sciences, Southern Medical University, Guangzhou, China; ^3^ Dongguan Key Laboratory of Respiratory and Critical Care Medicine, Affiliated Dongguan Hospital, Southern Medical University, Dongguan, China; ^4^ Department of Pediatrics, The Second Hospital of Zhuzhou, Zhuzhou, Hunan, China

**Keywords:** ring finger protein 169, tumour immune infiltration, pancreatic adenocarcinoma, non-coding RNA, bioinformatics

## Abstract

**Background:** Pancreatic adenocarcinoma (PAAD) is a highly deadly and aggressive tumour with a poor prognosis. However, the prognostic value of RNF169 and its related mechanisms in PAAD have not been elucidated. In this study, we aimed to explore prognosis-related genes, especially RNF169 in PAAD and to identify novel potential prognostic predictors of PAAD.

**Methods:** The GEPIA and UALCAN databases were used to investigate the expression and prognostic value of RNF169 in PAAD. The correlation between RNF169 expression and immune infiltration was determined by using TIMER and TISIDB. Correlation analysis with starBase was performed to identify a potential regulatory axis of lncRNA-miRNA-RNF169.

**Results:** The data showed that the level of RNF169 mRNA expression in PAAD tissues was higher than that in normal tissues. High RNF169 expression was correlated with poor prognosis in PAAD. In addition, analysis with the TISIDB and TIMER databases revealed that RNF169 expression was positively correlated with tumour immune infiltration in PAAD. Correlation analysis suggested that the long non-coding RNA (lncRNA) AL049555.1 and the microRNA (miRNA) hsa-miR-324-5p were involved in the expression of RNF169, composing a potential regulatory axis to control the progression of PAAD. Gene Ontology (GO) and Kyoto Encyclopedia of Genes and Genomes (KEGG) enrichment analyses indicated that RNF169 plays a role in PAAD through pathways such as TNF, Hippo, JAK-STAT and Toll-like receptor signaling.

**Conclusion:** In summary, the upregulation of RNF169 expression mediated by ncRNAs might influence immune cell infiltration in the microenvironment; thus, it can be used as a prognostic biomarker and a potential therapeutic target in PAAD.

## Introduction

Pancreatic adenocarcinoma (PAAD) is a deadly malignant tumour that is characterized by high mortality and poor prognosis (Parekh et al., 2017). In recent years, immunotherapy and targeted therapy strategies have been applied in the treatment of PAAD; however, the 5-year overall survival of PAAD patients has not significantly improved over the past few decades, highlighting the complexity of this disease (Morrison et al., 2018; Neoptolemos et al., 2018; Li et al., 2019). The molecular mechanisms underlying the formation and progression of tumours are not fully understood, which further complicates the effective treatment of PAAD. PAAD has an insidious onset and currently lacks biomarkers for early diagnosis (Zhou et al., 2021). Therefore, the identification of novel independent prognostic biomarkers is still of great significance for PAAD patients to improve the clinical outcome of this disease.

Ring finger protein 169 (RNF169) is an E3 ubiquitin-protein ligase that acts as a negative regulator of double-strand break (DSB) repair following DNA damage (Poulsen et al., 2012). RNF169 competes for RNF168-catalysed ubiquitin adducts and replaces TP53BP1 and RAP80 from DSBs (Chen et al., 2012; Panier et al., 2012; Poulsen et al., 2012). Previous studies showed that RNF169 suppresses TP53BP1 recruitment to chromatin ubiquitylated by RNF168 (Panier et al., 2012). RNF169 and USP7 expression was positively correlated in breast cancer samples (An et al., 2017). However, an integrative analysis of the expression, prognosis, immune infiltration and related mechanism of RNF169 in PAAD has not been performed.

It has been reported that 70%–90% of the human genome is transcribed into RNA, but 98%–99% of the human genome contains non-coding DNA, including long non-coding RNAs (lncRNAs), circular RNAs, pseudogenes and microRNAs (miRNAs) (Consortium, 2012). Many studies have shown that protein-coding mRNAs and non-coding RNAs (ncRNAs) play key roles in many important biological processes, including cell differentiation, gene expression, epithelial-mesenchymal transition (EMT), cell cycle arrest, apoptosis, and cell migration and invasion (Song et al., 2016; Song et al., 2018). Previous findings have demonstrated that lncRNAs are involved in PAAD (Qi et al., 2021). Elevated expression of miRNAs was reported to accelerate tumorigenesis and has been utilized in tumour diagnosis (Zhang et al., 2014). Previous findings have also demonstrated that lncRNAs competitively bind to target mRNAs against miRNAs and influence tumour occurrence and development (Wang et al., 2019).

The tumour immune microenvironment (TIME) comprises a series of infiltrating cells, including stromal cells, endothelial cells and immune cells (Yu et al., 2020). Accumulating evidence supports the conception that the TIME plays an important role in determining the therapeutic response to anticancer treatment as well as the tumour immune response (Wang et al., 2012; Takenaka et al., 2019). The immune components in the tumour microenvironment play critical roles in tumour progression, impacting tumour growth, metastasis and the response to cancer therapy (Liu et al., 2018). A key component of the tumour microenvironment motivating cellular immune responses is the cytokine/chemokine milieu (Karagiannidis et al., 2020). Cytokines and chemokines in the tumour microenvironment have been shown to promote breast cancer progression and metastasis (Venmar et al., 2014). However, the relationship between RNF169 and the TIME remains unclarified in PAAD.

In the present study, we explored the mRNA expression and prognostic value of RNF169 in PAAD based on GEPIA database analysis. The regulation of RNF169 associated with ncRNAs, including lncRNAs and miRNAs, was investigated in PAAD. We also utilized Kyoto Encyclopedia of Genes and Genomes (KEGG) and Gene Ontology (GO) enrichment analyses to explore the functions of coexpression networks with RNF169 to explain the potential mechanisms of PAAD. We found a potential correlation between immune infiltration and the expression of RNF169 in the microenvironment of PAAD by using the TISIDB and TIMER databases. Our results suggest that ncRNA-mediated RNF169 upregulation is associated with poor prognosis and tumour immune infiltration in patients with PAAD.

## Materials and methods

### GEPIA analysis

Gene Expression Profiling Interactive Analysis (GEPIA, http://gepia.cancer-pku.cn/index.html) Tang et al. (2017) is a web tool for the analysis of tumour and adjacent normal samples. GEPIA provides a series of key customizable and interactive functions, including differential expression analysis, correlation gene analysis, similar gene detection, survival analysis, and profiling plotting analysis. GEPIA was utilized to explore the differential expression of RNF169 in tumour and normal tissues. We compared the expression patterns of RNF169 in 30 different types of tumours. GEPIA was applied to estimate associations of RNF169 expression with the overall survival (OS) and disease-free survival (DFS) of PAAD patients. GEPIA was also used to perform the stage plot evaluation and analysis of the correlation between RNF169 expression and infiltration of immune cells.

### LinkedOmics analysis

LinkedOmics (http://linkedomics.org/login.php) is a publicly available web tool that contains multiomics data from all 32 TCGA cancer types (Vasaikar et al., 2018). The LinkCompare module provides an easy comparison of the associations verified by LinkFinder, which is especially useful in multiomics and pan-cancer research. The linkInterpreter module of LinkedOmics was used to derive biological insights from coexpressed gene enrichment by using the Spearman correlation test. The genes correlated with RNF169 expression are shown in a volcano plot, and some representative genes are displayed in scatter plots. Gene set enrichment analysis (GSEA) was used to perform Gene Ontology (GO) and Kyoto Encyclopedia of Genes and Genomes (KEGG) pathway analyses (Kanehisa et al., 2021).

### TIMER database analysis

The TIMER database (https://cistrome.shinyapps.io/timer/) (Li et al., 2017) is a web database for evaluating the relationships between clinical associations, somatic copy number alterations (SCNAs), and mutations and the infiltration of different immune cells, including neutrophils, macrophages, B cells, CD4^+^ T cells, CD8^+^ T cells and dendritic cells. To investigate the potential mechanisms of RNF169 in the regulation of tumour-infiltrating immune cells, we utilized the TIMER database to determine the correlation between RNF169 expression and immune infiltrating cells in PAAD. The correlation coefficient was evaluated by using the Spearman’s correlation method. The SCNA module in the TIMER database was used to evaluate the relationship between RNF169 copy number variations (CNVs) and immune cell infiltration.

### UALCAN database analysis

UALCAN (http://ualcan.path.uab.edu) is a comprehensive, user-friendly and interactive web resource for analysing cancer omics data (Chandrashekar et al., 2017). In the present study, we used the UALCAN database to evaluate the effect of RNF169 expression on the survival of PAAD patients.

### TISIDB analysis

TISIDB (http://cis.hku.hk/TISIDB) is a publicly available web portal that includes a summary of 988 immune-related antitumour genes for 30 cancer types from the TCGA (Ru et al., 2019; Wang et al., 2021). TISIDB provides a tool to analyse the interaction of certain genes with lymphocytes, immunomodulators and chemokines. In the present study, this database was utilized to explore the correlation between RNF169 expression and lymphocytes.

### StarBase database analysis

StarBase (http://starbase.sysu.edu.cn/) is a database tool for miRNA-related studies (Li et al., 2014). In the present study, starBase was utilized to analyse the interaction between miRNAs and their targeted lncRNAs and to determine the prognostic value of miRNA expression in PAAD.

### Immunohistochemistry (IHC) staining

Human pancreatic tissue samples were used to detect the protein expression of RNF169 by immunohistochemistry. Tissues were fixed with 4% paraformaldehyde and embedded in paraffin. The sections were dewaxed and heated in the improved citrate antigen retrieval solution for antigen repair and permeabilized with immunostaining permeabilization buffer with Triton X-100. Endogenous peroxidase was blocked with endogenous peroxidase blocking buffer. The sections were incubated with a specific primary antibody overnight at 4°C, followed by detection with an HRP-labeled secondary antibody. The images were visualized with an automatic immunohistochemistry instrument from DAKO (Carpinteria, CA, United States). Samples were counterstained with hematoxylin for 1 min, and incubated in ethanol and xylene solution of ascending concentration. The sections were finally sealed with neutral resins.

### Statistical analysis

The statistical analysis was performed by using the online databases mentioned above. A *p*-value less than .05 was considered statistically significant.

## Results

### RNF169 expression in multiple human cancers

**FIGURE 1:**
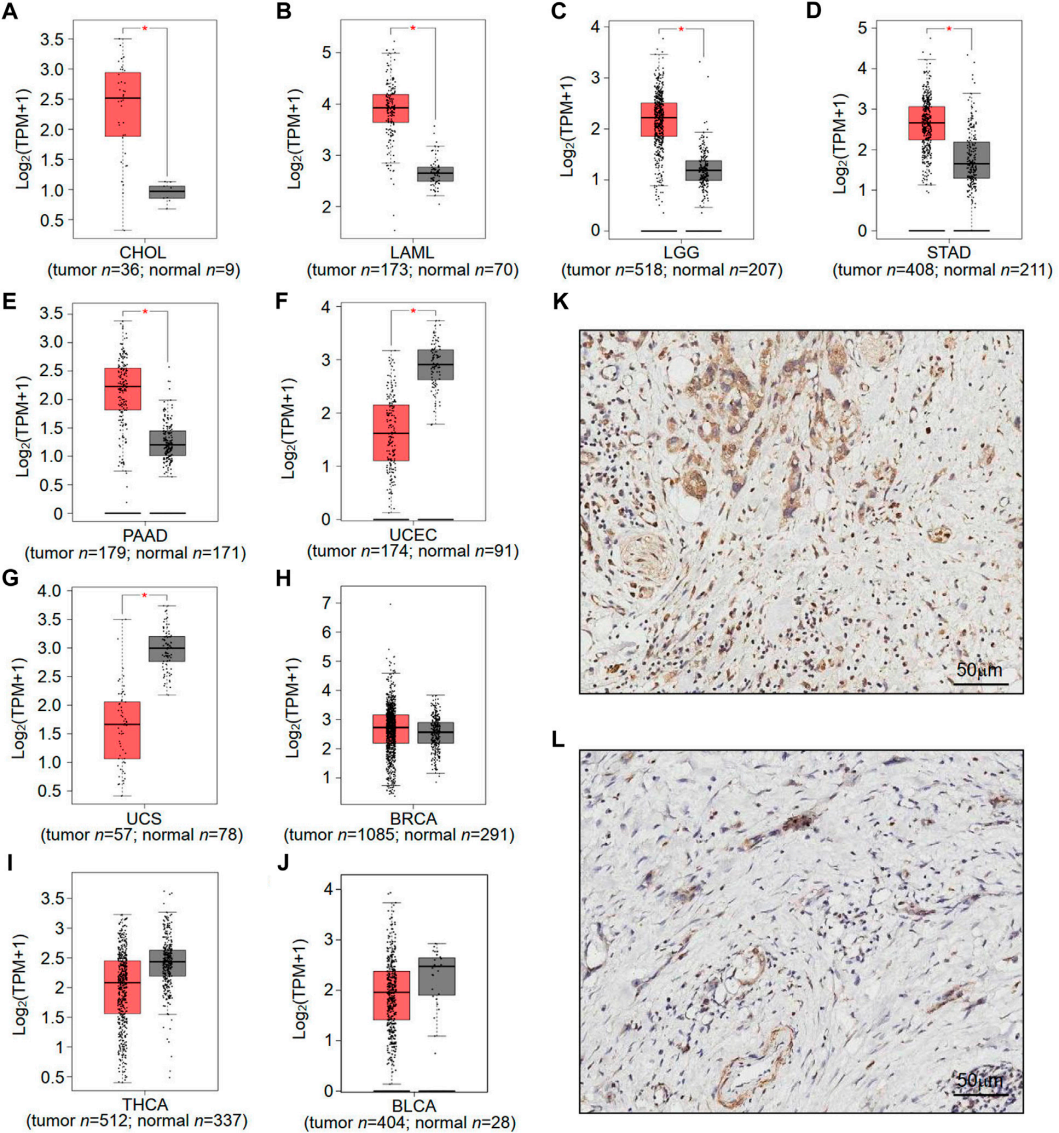
Differential expression of RNF169 in cancer and normal tissues. **(A–J)** RNF169 expression in 10 different types of human cancers in the GEPIA database, including CHOL **(A)**, LAML **(B)**, LGG **(C)**, STAD **(D)**, PAAD **(E)**, UCEC **(F)**, UCS **(G)**, BRCA **(H)**, THCA **(I)** and BLCA **(J)**,and in comparison with adjacent normal tissues. (*p < .05, **p < .01, ***p < .001). **(K,L)** Representative immunohistochemical staining of RNF169 in PAAD and tumor-adjacent tissues. Immunohistochemical staining for RNF169 was performed in PAAD **(K)** and tumor-adjacent tissues **(L)**.

To understand the potential role of RNF169 in carcinogenesis, we sought to investigate its expression in different human tumours. We utilized the GEPIA database to analyse the expression levels of RNF169 in normal and tumour tissues from the Genotype-Tissue Expression (GTEx) project and TCGA and found that there was a significant increase in RNF169 expression in cholangiocarcinoma (CHOL), acute myeloid leukemia (LAML), brain lower grade glioma (LGG), stomach adenocarcinoma (STAD) and pancreatic adenocarcinoma (PAAD) tissues compared with normal tissues (Figures 1A–E). In contrast, the expression of RNF169 was significantly decreased in uterine corpus endometrial carcinoma (UCEC) and uterine carcinosarcoma (UCS) tissues compared with normal tissues (Figures 1F, G). However, we found that there was no difference in the expression of RNF169 between normal and tumour tissues including breast cancer (BRCA), thyroid carcinoma (THCA), bladder urothelial carcinoma (BLCA) (Figure 1H), cervical squamous cell carcinoma (CESC), colon adenocarcinoma (COAD), esophageal carcinoma (ESCA), glioblastoma multiforme (GBM), head and neck squamous cell carcinoma (HNSC), sarcoma (SARC), kidney renal clear cell carcinoma (KIRC), testicular germ cell tumour (TGCT), liver hepatocellular carcinoma (LIHC), lung adenocarcinoma (LUAD), skin cutaneous melanoma (SKCM), ovarian serous cystadenocarcinoma (OV), thymoma (THYM), prostate adenocarcinoma (PRAD), rectum adenocarcinoma (READ), adrenocortical carcinoma (ACC), kidney renal papillary cell carcinoma (KIRP), kidney chromophobe (KICH), lung squamous cell carcinoma (LUSC), and pheochromocytoma and paraganglioma (PCPG) (Supplementary Figure S1). In order to demonstrate whether RNF169 plays a role in the patients, we performed immunohistochemical staining of RNF169 in human tissues and found that RNF169 was elevated in PAAD tissues compared with adjacent tissues (Figures 1K, L)

### Prognostic value of RNF169 in multiple human cancers

**FIGURE 2:**
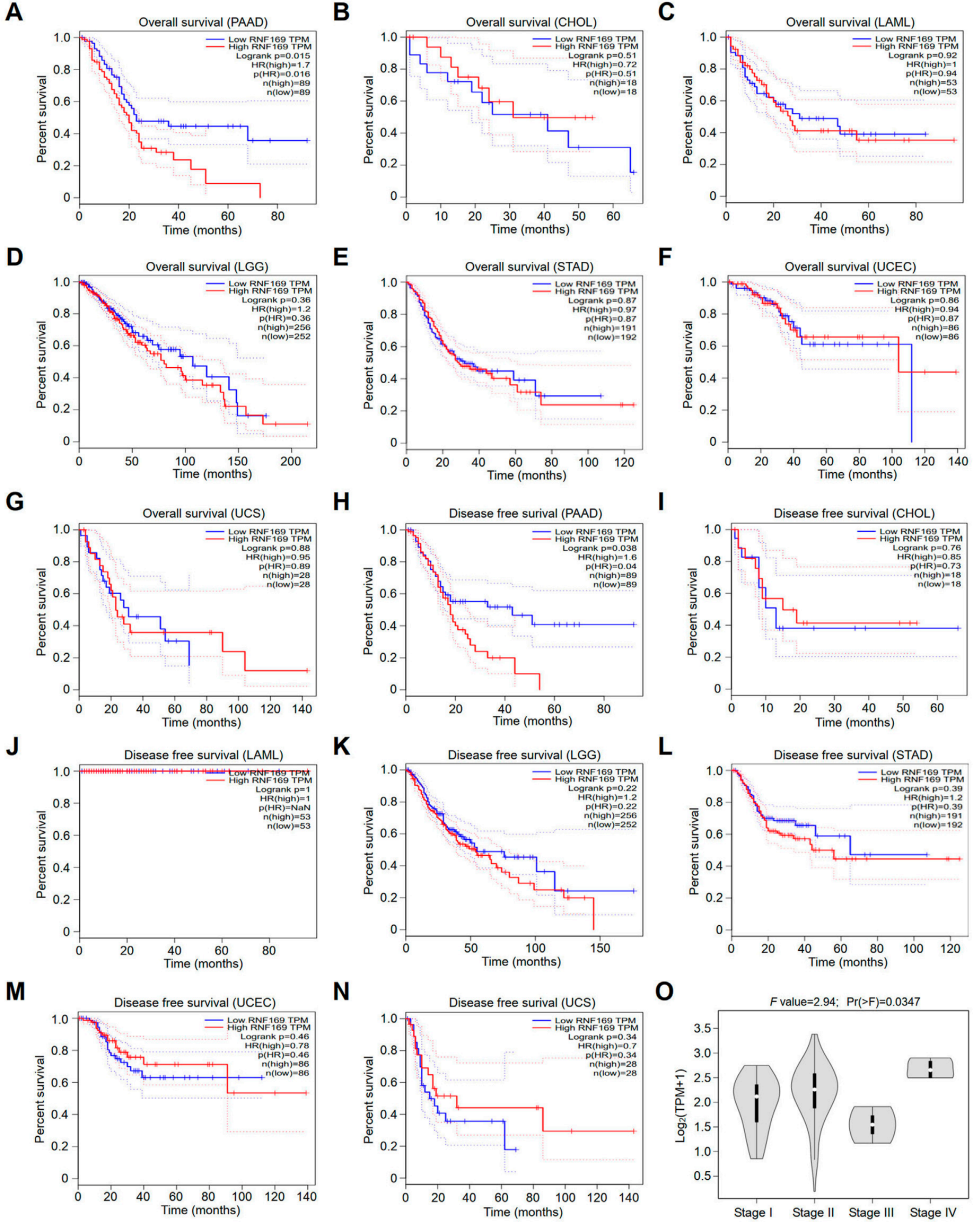
Survival analyses of patients with different cancers with high or low RNF169 expression. **(A–G)** The overall survival (OS) plot of patients with high or low RNF169 expression in PAAD **(A)**, CHOL **(B)**, LAML **(C)**, LGG **(D)**, STAD **(E)**, UCEC **(F)** and UCS **(G)**. **(H–N)** Disease-free survival (RFS) analysis for patients with high or low RNF169 expression in different cancers, i.e., PAAD **(H)**, CHOL **(I)**, LAML **(J)**, LGG **(K)**, STAD **(L)**, UCEC **(M)** and UCS **(N)**. **(O)** Correlation of tumour progression with RNF169 mRNA expression in PAAD patients from the GEPIA database.

Next, we compared the overall survival (OS) and disease-free survival (DFS) of patients in terms of RNF169 expression levels to analyse the prognostic value of RNF169 across cancers. Interestingly, we found that high expression of RNF169 was an unfavourable indicator for the OS of patients with PAAD (Figure 2A). In contrast, RNF169 expression did not show significance for evaluating the prognosis of patients with other cancer types (Figures 2B–G). In addition, upregulation of RNF169 expression was linked to poor DFS in PAAD patients (Figure 2H) but was not associated with the outcome of other cancer patients (Figures 2I–N). To determine the clinical relevance of RNF169 in PAAD, we used the GEPIA database to analyse the correlation of RNF169 expression with different tumour stages and found that there was a significant correlation between them (Figure 2O). To further verify the results above, we performed survival analysis with the TIMER (Supplementary Figure S2A) and UALCAN (Supplementary Figure S2B) databases and found that PAAD patients with lower RNF169 expression had better cumulative survival. The clinicopathological characteristics were analyzed by the Wilxcon test. Notably, a significant correlation of RNF169 expression was observed with age (*p* < .05), but not other clinical traits (T, N, M and stage) (Supplementary Figure S3). Then, we had constructed a TCGA cohort consisting of age, grade, stage and RNF169 expression and the results of multivariate Cox regression analysis (Xu et al., 2022) showed that the RNF169 expression was an independent factor capable of predicting poor survival (Supplementary Figure S4).

### Gene mutation information of RNF169 in PAAD

Previous genomic studies have demonstrated that genetic mutations play an important role in pancreatic cancer development (Liu et al., 2021). The “maftools” package in R software was utilized to analyse the gene mutation rate in PAAD from the TCGA database. The results showed that, unlike the representative oncogenes including *KRAS*, *TP53*, *SMAD4*, *CDKN2A, TTN, MUC16, RNF43, GNAS, RYR1, and ARID1A* with multiple mutations*,* RNF169 was only detected with a single missense mutation with a frequency of 1% (Supplementary Figure S5A). Of all 178 samples, 145 PAAD patients were identified to have missense mutations, which represents the most frequent mutation type in PAAD patients (Supplementary Figure S5B). We found that single nucleotide polymorphisms (SNPs) accounted for the majority of variant types, which was much higher than the number of insertions and deletions, and the C>T mutation represented 60.08% of single nucleotide variants (SNVs) in PAAD patients (Supplementary Figure S5C). Statistical analysis showed that the median number of variants per PAAD sample was approximately 33.5 (Supplementary Figure S5D), and missense mutations accounted for approximately 44% of the variant classes (Supplementary Figure S5E). For the top 10 mutated genes in PAAD, including *KRAS, TP53, TTN, SMAD4, MUC16, CDKN2A, RYR1, RNF43, ARID1A and GNAS,* missense mutations occurred with the highest frequency (Supplementary Figure S5F).

### Correlation of RNF169 expression with cytokines, chemokines, and chemokine receptors

**FIGURE 3:**
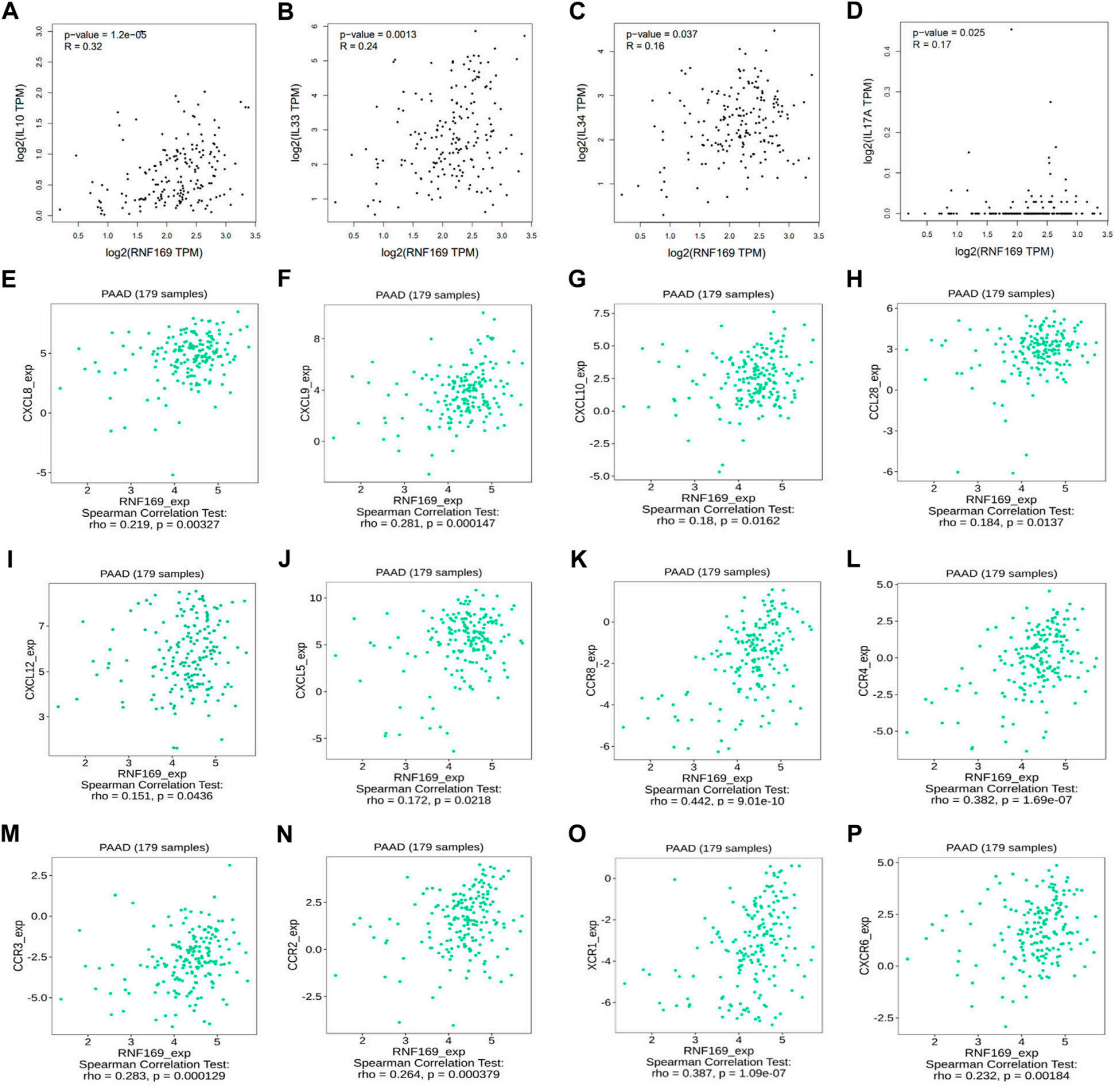
Correlation of RNF169 expression with cytokines, chemokines, chemokine receptors in PAAD. **(A–D)** The correlation of RNF169 expression with various cytokines. **(E–J)** The correlation of RNF169 expression with various chemokines. **(K–P)** The correlation of RNF169 expression with chemokine receptors.

Over the years, several cytokines have been identified as key promoters of cancer progression and metastasis development (Yao et al., 2014). Our results showed that the expression of RNF169 was positively correlated with Interleukin 10 (IL-10), IL-33, IL-34, and IL-17A (Figures 3A–D). Chemokines and their receptors play key roles in the crosstalk between the tumour microenvironment (TME) and cancer cells and are involved in the regulation of tumour immune cell infiltration, angiogenesis, growth, invasion and metastasis (Portella et al., 2021). We examined the correlations between RNF169 expression and chemokines and chemokine receptors in the TISIDB database and found that several chemokines (Figures 3E–J) and chemokine receptors (Figures 3K–P) have significant correlations with RNF169 expression in PAAD. These results support the conclusion that RNF169 is positively correlated with multiple cytokines, chemokines and chemokine receptors.

### Correlation between RNF169 expression and infiltrated immune cells in PAAD

**FIGURE 4:**
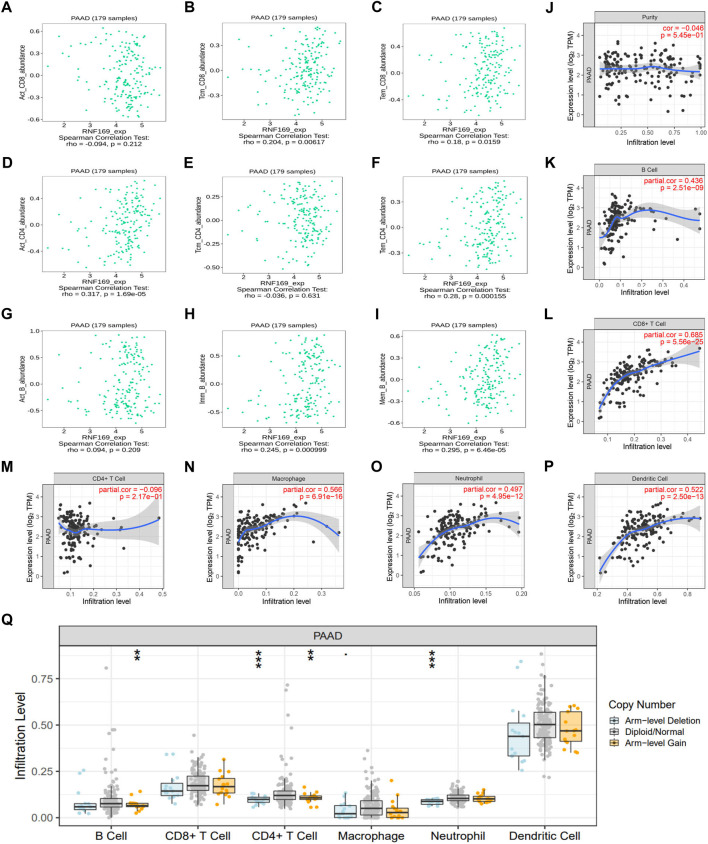
Correlation of RNF169 expression with different infiltrated immune cells in PAAD. **(A–C)** Correlation of RNF169 expression with the number of activated CD8^+^ T cells **(A)**, central memory CD8^+^ T cells **(B)** and effector memory CD8^+^ T cells **(C)** in PAAD from the TISIDB database. **(D–F)** Correlations of RNF169 expression with the number of activated CD4^+^ T cells **(D)**, central memory CD4^+^ T cells **(E)** and effector memory CD4^+^ T cells **(F)** in PAAD. **(G–I)** Correlations of RNF169 expression with the number of activated B cells **(G)**, immature B cells **(H)** and memory B cells **(I)** in PAAD. **(J)** Relationship between RNF169 expression and tumour purity in PAAD from the TIMER database. **(K–P)** Correlations of RNF169 expression with the number of different infiltrated immune cells, including B cells **(K)**, CD8^+^ T cells **(L)**, CD4^+^ T cells **(M)**, macrophages **(N)**, neutrophils **(O)** and dendritic cells **(P)**, in PAAD from the TIMER database. **(Q)** Effect of RNF169 CNV on the infiltration of B cells, CD8^+^ T cells, CD4^+^ T cells, macrophages, neutrophils and dendritic cells in PAAD.

Previous studies have shown that the infiltration of cancer-related immune cells is associated with tumour progression and prognosis (Yan et al., 2021). Based on the results above regarding cytokines, chemokines and chemokine receptors, we further analysed the correlation between RNF169 mRNA expression and immune cell infiltration in tumours. We found that the expression of RNF169 was significantly correlated with the infiltration of some T and B cells, including central memory CD8^+^ T cells (*r* = .204, *p* = .00617) (Figure 4B), effector memory CD8^+^ T cells (*r* = .18, *p* = .0159) (Figure 4C), activated CD4^+^ T cells (*r* = .317, *p* = 1.69 e−05) (Figure 4D), effector memory CD4^+^ T cells (*r* = .28, *p* = .000155) (Figure 4F), immature B cells (*r* = .245, *p* = .000999) (Figure 4H) and memory B cells (*r* = .295, *p* = 6.46 e−05) (Figure 4I), but not activated CD8^+^ T cells (*r* = −.094, *p* = .212) (Figure 4A), central memory CD4^+^ T cells (*r* = −.036, *p* = .631) (Figure 4E) or activated B cells (*r* = .094, *p* = .209) (Figure 4G). Although RNF169 expression had no significant correlation with tumour purity (*r* = −.046, *p* = 5.45 e−01) (Figure 4J), we observed a significant correlation between RNF169 expression and the infiltration levels of B cells (*r* = .436, *p* = 2.51e−09) (Figure 4K), CD8^+^ T cells (*r* = .685, *p* = 5.56 e−25) (Figure 4L), macrophages (*r* = .566, *p* = 6.91 e−16) (Figure 4N), neutrophils (*r* = .497, *p* = 4.95 e−12) (Figure 4O) and dendritic cells (DCs) (*r* = .522, *p* = 2.50 e−13) (Figure 4P) in PAAD, but not CD4^+^ T cells (*r* = −.096, *p* = 2.17 e−01) (Figure 4M).

Then, we investigated the relationship between somatic copy number alterations of RNF169 and the abundance of immune infiltrates in PAAD and found that the copy number variation (CNV) of RNF169 was significantly associated with the infiltration levels of B cells, CD4^+^ T cells and neutrophils (Figure 4Q). These findings suggest that RNF169 could serve as a major tumour immune infiltration regulator in PAAD. We performed TIDE analysis and found an obvious positive correlation between RNF169-associated risk and TIDE score (Supplementary Figure S6).

### Correlation between RNF169 expression and markers of infiltrated immune cells in PAAD

**FIGURE 5:**
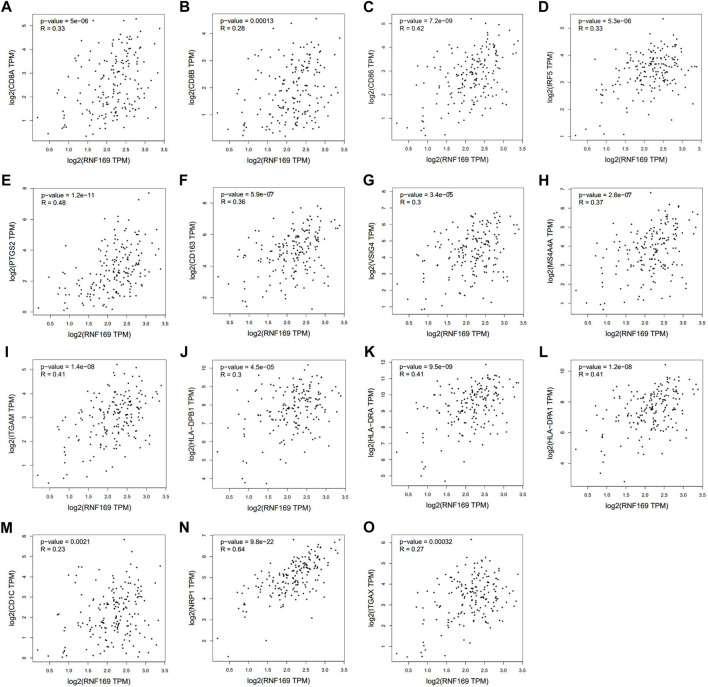
Correlations of RNF169 expression with biomarkers of different immune cells. Correlation analysis of RNF169 expression with different immune cell biomarkers was performed with the GEPIA database. **(A,B)** Correlations of RNF169 expression with the CD8^+^ T cell biomarkers CD8A **(A)** and CD8B **(B)**. **(C)** Correlations of RNF169 expression with the costimulatory molecule CD86. **(D,E)** Correlations of RNF169 expression with the M1 macrophage biomarkers IRF5 **(D)** and PTGS2 **(E)**. **(F–H)** Correlations of RNF169 expression with the M2 macrophage biomarkers CD163 **(F)**, VSIG4 **(G)** and MS4A4A **(H)**. **(I)** Correlations of RNF169 expression with the neutrophil biomarker ITGAM. **(J–O)** Correlations of RNF169 expression with the dendritic cell biomarkers HLA-DPB1 **(J)**, HLA-DRA **(K)**, HLA-DPA1 **(L)**, CD1C **(M)**, NRP1 **(N)** and ITGAX **(O)** in PAAD.

We further used the GEPIA database to explore the relationship between RNF169 and different immune markers of immune cells in PAAD. We observed a significant positive correlation between RNF169 expression and the levels of CD8^+^ T cell biomarkers (CD8A and CD8B), costimulatory molecule (CD86), M1 macrophage biomarkers, such as Interferon regulatory factor 5 (IRF5) and prostaglandin-endoperoxide synthase 2 (PTGS2), M2 macrophage biomarkers, such as CD163, V-set and Ig domain-containing 4 (VSIG4) and membrane spanning 4-domains A4A (MS4A4A), neutrophil biomarkers (integrin subunit alpha M, ITGAM) and dendritic cell biomarkers, such as HLA class II histocompatibility antigen, DP beta 1 chain (HLA-DPB1), HLA class II histocompatibility antigen, DR alpha chain (HLA-DRA), HLA class II histocompatibility antigen, DP alpha 1 chain (HLA-DPA1), CD1C, neuropilin 1 (NRP1) and integrin subunit alpha X (ITGAX) (Figure 5A–O), which reveals that RNF169 participates in the regulation of tumour immune infiltration in PAAD.

### Correlation of RNF169 expression with immune checkpoints in PAAD

**FIGURE 6:**
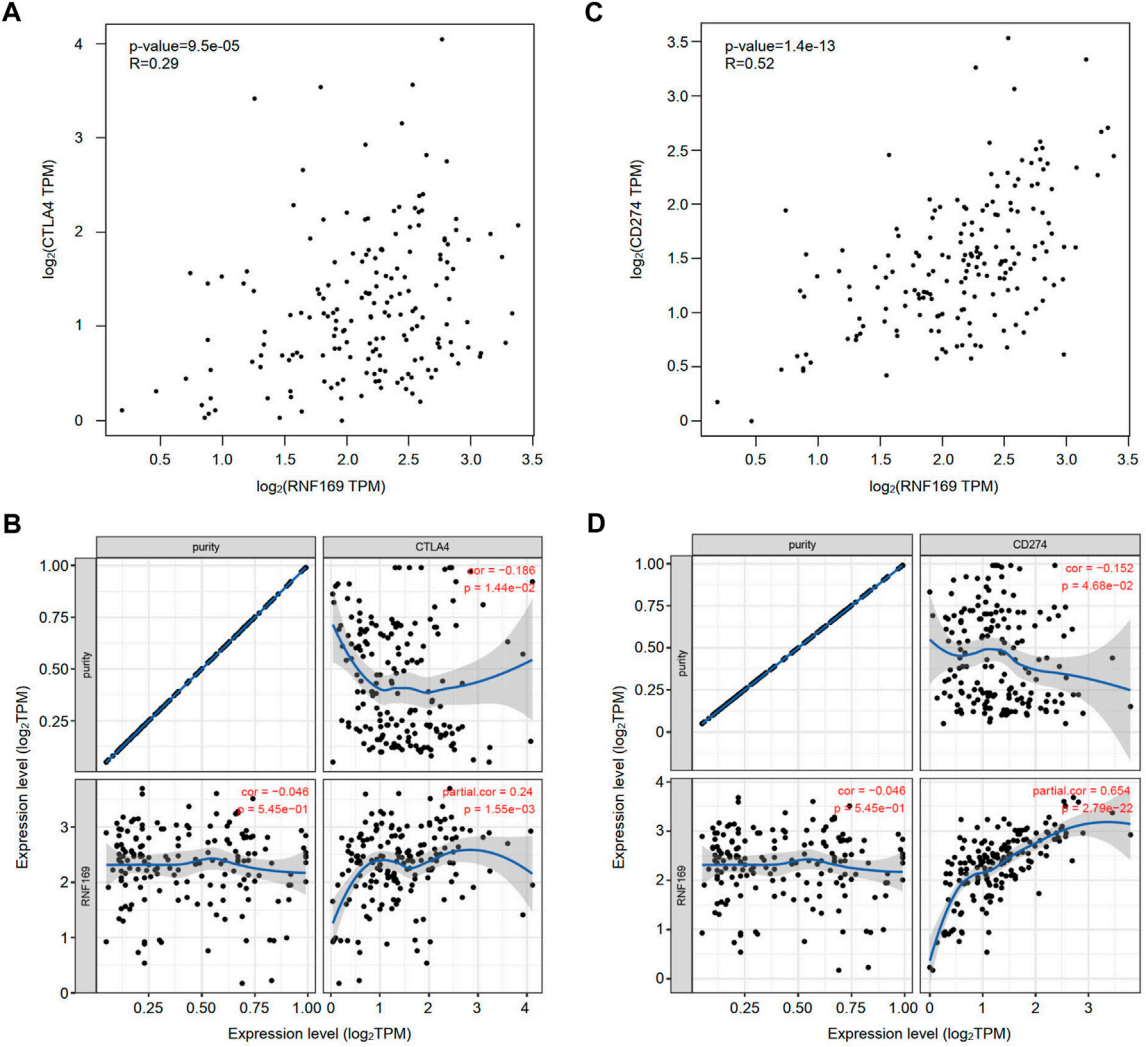
Correlation of RNF169 with the expression of CTLA4 or CD274 in PAAD. **(A,B)** The correlation of RNF169 with CTLA4 in PAAD was analysed by using the GEPIA **(A)** and TIMER **(B)** databases. **(C,D)** The correlation of RNF169 with CD274 in PAAD was analysed by using the GEPIA **(C)** and TIMER **(D)** databases.

Immune checkpoint molecules, including cytotoxic T-lymphocyte-associated protein 4 (CTLA4), programmed cell death protein-1 (PD-1) and programmed cell death ligand-1 (PD-L1), are responsible for tumour escape from the immune response (Lee et al., 2019). Considering the potential oncogenic role of RNF169 in PAAD development, we evaluated the correlation of RNF169 with CTLA4 and CD274. We found that RNF169 expression was positively correlated with CTLA4 (Figures 6A, B) and CD274 (Figures 6C, D) in PAAD from the GEPIA and TIMER databases. These results indicate that tumour immune escape might be involved in the RNF169-induced carcinogenesis of PAAD.

### RNF169 gene coexpression profiles and KEGG and GO enrichment analyses in PAAD

**FIGURE 7:**
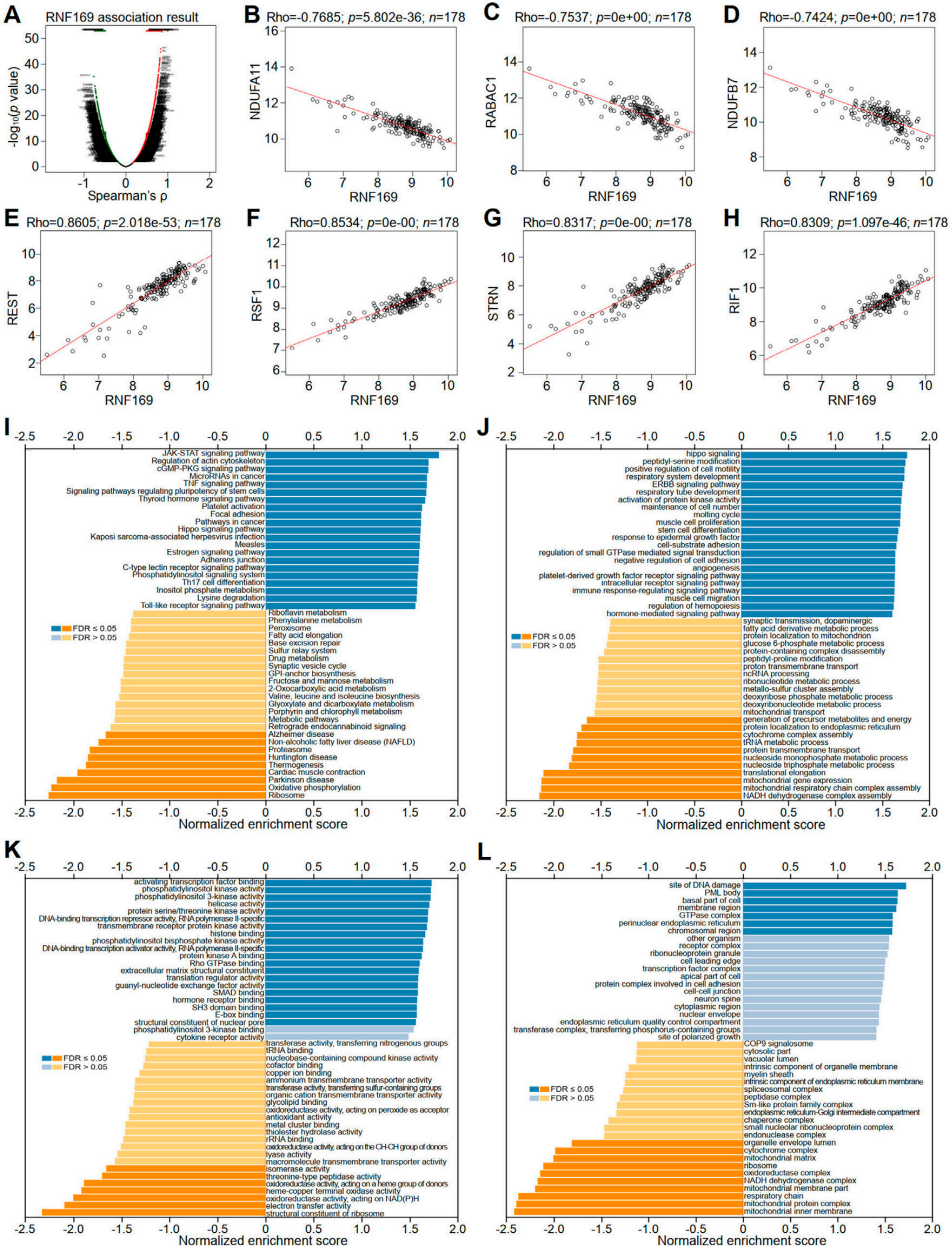
Correlation of RNF169 with the expression of differentially expressed genes in PAAD. **(A)** Volcano plots showing the differentially expressed genes in PAAD. **(B–D)** Negative correlation of RNF169 with three representative DEGs, i.e., NDUFA11 **(B)**, RABAC1 **(C)** and NDUFB7 **(D)**, in PAAD. **(E–H)** Positive correlation of RNF169 with four representative DEGs, i.e., REST **(E)**, RSF1 **(F)**, STRN **(G)**, RIF1 **(H)**, in PAAD. **(I)** KEGG pathway analysis of DEGs in PAAD. **(J–L)** GO term enrichment of biological processes **(J)**, molecular functions **(K)** and cellular components **(L)** was analysed by GSEA.

To better understand the role of RNF169 in the progression of PAAD, LinkedOmics was utilized to investigate its coexpressed genes. A volcano plot was used to display the differentially expressed genes correlated with the RNF169 gene in PAAD (Figure 7A). Several representative genes with negative (Figures 7B–D) or positive (Figures 7E–H) correlations with RNF169 are shown in Figure 7. The LinkInterpreter module was used for Kyoto Encyclopedia of Genes and Genomes (KEGG) pathway analysis of genes coexpressed with RNF169, which showed that the coexpressed genes in PAAD are involved in the Janus kinase (JAK)-signal transducer of activators of transcription (STAT) signaling pathway, tumour necrosis factor (TNF) signaling pathway, Toll-like receptor signaling pathway and pathways in cancer (Figure 7I). Gene Ontology (GO) analysis showed that the biological process terms were mainly enriched in the Hippo signaling, positive regulation of cell motility and immune response-regulating signaling pathway (Figure 7J); the molecular function terms were mainly enriched in phosphatidylinositol 3-kinase (PI3K) activity, DNA-binding transcription repressor activity, RNA polymerase II-specific and Rho GTPase binding (Figure 7K); and the cellular component terms were mainly enriched in membrane region and perinuclear endoplasmic reticulum (Figure 7L). These findings indicate that RNF169 participates in the JAK-STAT, TNF, and Toll-like receptor signaling pathways to promote tumorigenesis of PAAD.

We further performed GSEA analysis and found that RNF169 was involved in the regulation of inflammation and immune pathways, including RIG-1-like receptor pathway, Toll-like receptor signaling pathway, cytosolic DNA sensing pathway, steroid hormone biosynthesis, regulation of autophagy and autoimmune thyroid disease pathways (Supplementary Figure S7).

### Prediction and validation of potential miRNAs targeting RNF169

**FIGURE 8:**
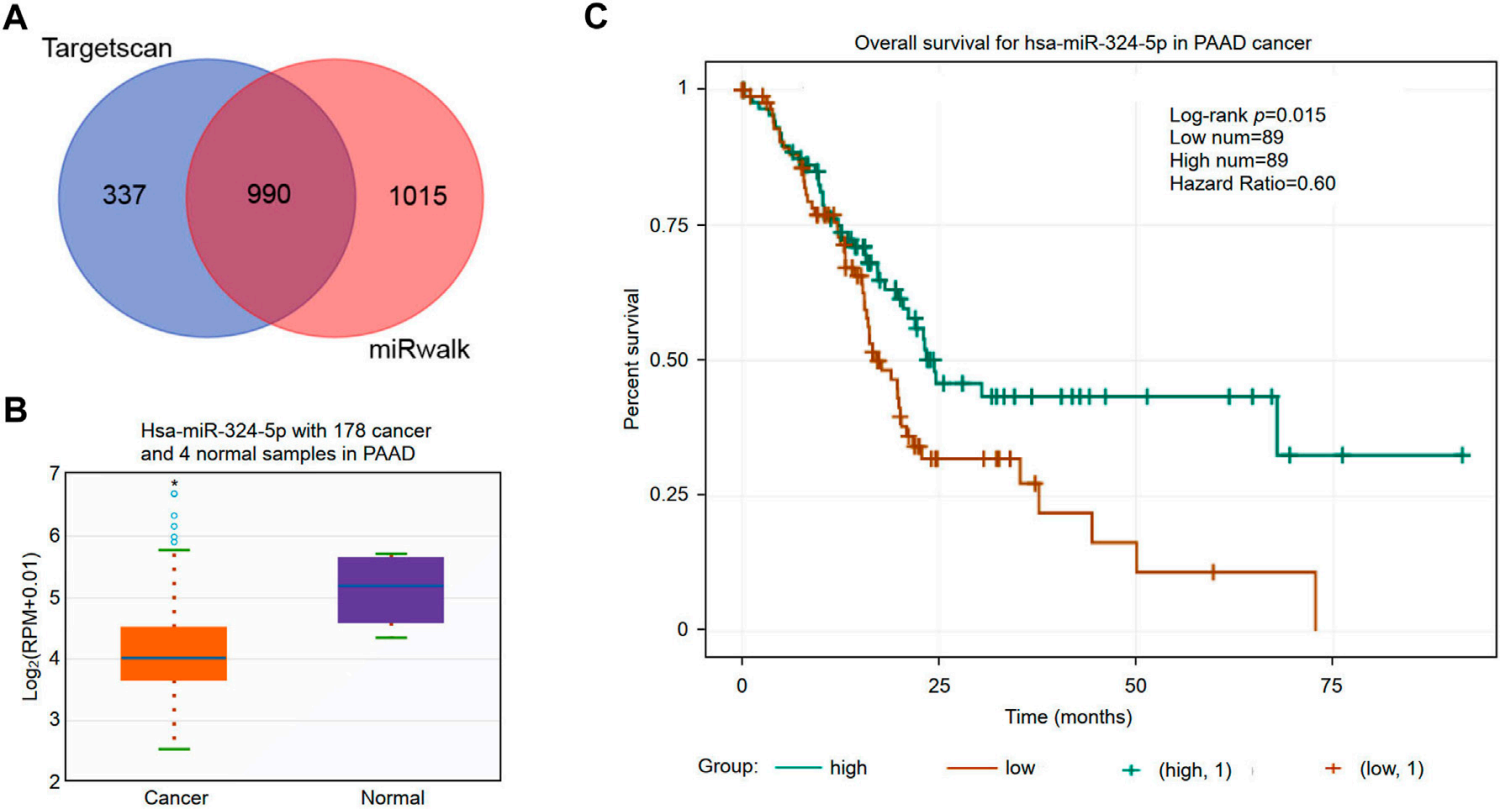
Identification of potential miRNAs associated with the prognosis of PAAD. **(A)** Identification of potential miRNAs targeting the RNF169 gene in the miRWalk and TargetScan databases. **(B)** Expression of hsa-miR-324-5p in PAAD and adjacent normal tissues. **(C)** Prognostic value of hsa-miR-324-5p for PAAD patients.

Non-coding RNAs (ncRNAs) participate in the regulation of target gene function in mammalian cells (Garding et al., 2013). Using the miRWalk and TargetScan databases, we obtained a total of 990 predicted miRNAs targeted to the RNF169 gene in PAAD (Figure 8A). Based on the mechanism of action of miRNAs in the regulation of target gene expression, we performed correlation analysis of the gene expression of RNF169 with these miRNAs. As shown in Figure 8B, hsa-miR-324-5p was significantly downregulated in PAAD compared with adjacent normal samples. We evaluated the prognostic value of hsa-miR-324-5p in PAAD and found that high expression of hsa-miR-324-5p was positively correlated with the prognosis of PAAD patients (Figure 8C), indicating that hsa-miR-324-5p is a potential regulatory miRNA for RNF169 in PAAD.

### Prediction and validation of the upstream lncRNAs of hsa-miR-324-5p

**FIGURE 9:**
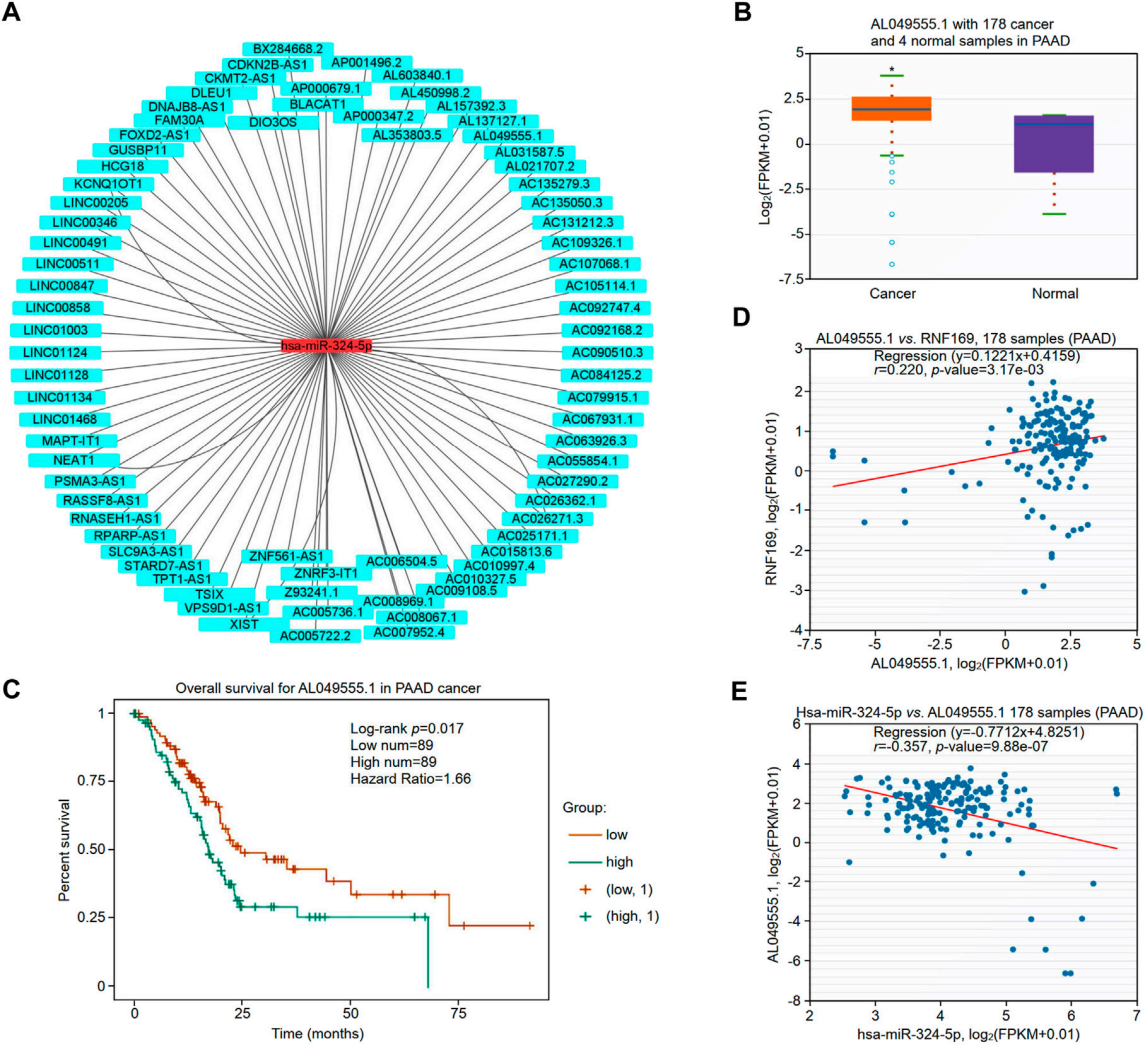
Validation of lncRNA AL049555.1 expression and its prognostic value in PAAD from the StarBase database. **(A)** The potential hsa-miR-324-5p-regulated lncRNA network constructed by Cytoscape. **(B)** Expression of lncRNA AL049555.1 in PAAD and adjacent normal tissues. **(C)** Prognostic value of lncRNA AL049555.1 for PAAD patients. **(D)** Coexpression analysis of RNF169 with the lncRNA AL049555.1 in PAAD patients. **(E)** Coexpression analysis of hsa-miR-324-5p with the lncRNA AL049555.1.

The upstream lncRNAs of hsa-miR-324-5p were predicted based on the starBase database, and a total of 77 potential lncRNAs were predicted. Next, Cytoscape software was utilized to construct the network of lncRNAs regulated by hsa-miR-324-5p (Figure 9A). The lncRNA AL049555.1 was significantly upregulated in PAAD compared with normal samples (Figure 9B), and PAAD patients with higher expression levels of AL049555.1 had poorer overall survival (Figure 9C). Previous studies have shown that ncRNAs act as competing endogenous RNAs (ceRNAs) to regulate the level of target mRNAs by competing with the miRNAs binding to the target mRNA (Choi et al., 2021). Correlation analysis showed a positive or negative correlation between AL049555.1 and RNF169 (Figure 9D) or hsa-miR-324-5p (Figure 9E). Overall, AL049555.1 might be the potential upstream lncRNA of the hsa-miR-324-5p/RNF169 axis in PAAD.

## Discussion

RNF169 is an E3 ubiquitin-protein ligase that maintains balance by limiting the deposition of DNA damage mediator proteins at damaged chromatin (An et al., 2017). RNF169 contains a RING finger motif and ubiquitin-binding modules (UIMs), an MIU-related ubiquitin binding domain (UMI) at the N-terminus, and a putative motif interacting with the ubiquitin (MIU) domain and an LR motif (LRM) at the C-terminus (Penengo et al., 2006; Panier et al., 2012). RNF169 acts as a negative regulator of double-strand break (DSB) repair following DNA damage by interacting with various partners. For example, the lncRNA PRLH1 was identified as an RNF169 interactor that promotes homologous recombination (HR) repair (Deng et al., 2019); RNF169 is a new interacting partner for dual-specificity tyrosine-phosphorylation-regulated kinase 1A (DYRK1A) (Roewenstrunk et al., 2019). RNF169 was also found to bind to ubiquitylated H2A-Lys13/Lys15 in a manner that involves its canonical ubiquitin-binding helix and a pair of arginine-rich motifs (Kitevski-LeBlanc et al., 2017). It was reported that the nuclear localization signal (NLS) not only determines the shuttling of RNF169 into the nucleus but also promotes its stability by mediating a direct interaction with the ubiquitin-specific protease USP7, indicating an NLS-mediated bipartite mechanism that supports the nuclear function of a DSB response protein. Many studies highlight a critical key role of RNF169 in genome stability maintenance (An et al., 2017); however, the mechanism underlying the function of RNF169 in orchestrating the response to DNA double-strand breaks (DSBs) in PAAD remains largely unknown.

Pancreatic adenocarcinoma (PAAD) is a lethal malignancy with a strong metastatic ability, high aggression and a poor prognosis (Rahib et al., 2014). It is necessary to investigate more effective prognostic markers and therapeutic targets. However, there is a lack of integrated bioinformatic analyses of RNF169 in PAAD. To address this issue, we conducted a comprehensive analysis of RNF169 to understand its effect on the progression and prognosis of PAAD. In this study, RNF169 was found to be upregulated in a variety of human carcinomas, including CHOL, LAML, LGG, STAD and PAAD, but downregulated in UCEC and UCS. These results suggest that RNF169 may act as a regulator in the carcinogenesis of these seven different tumours. Further analysis showed that high expression of RNF169 predicts worse OS and DFS in PAAD patients, which suggests that RNF169 may be used as an unfavourable prognostic biomarker in patients with PAAD.

lncRNAs and immunity are an emerging field that offers a new perspective on cancer immunity and immunotherapies for cancer (Marin-Bejar et al., 2017). lncRNAs have been demonstrated to modulate immune function (Zhang et al., 2021). Previous studies have found that lncRNA-based molecular subtyping offers valuable insights into the molecular landscape of tumours (Niknafs et al., 2016). In recent years, many studies have shown that ncRNAs are involved in the regulation of gene expression by crosstalk with other small RNAs, such as miRNAs, through ceRNA mechanisms (Ghafouri-Fard et al., 2020; Lou et al., 2020). To study the upstream miRNAs regulating RNF169, we utilized two online software programs to predict the potential miRNAs binding with near perfect complementarity to target RNF169 and acquired 990 miRNAs by overlapping the results from miRWalk and TargetScan. It has been reported that overexpression of hsa-miR-324-5p significantly suppresses cell proliferation in colorectal cancer cells (Gu et al., 2019). Another study showed that the lncRNA LINC00511 increases proliferation and invasion by sponging hsa-miR-324-5p to regulate DRAM1 expression in cervical cancer cells (Zhang X. et al., 2020). Based on the ceRNA theory for gene regulation, it is reasonable to explore the oncogenic role of lncRNAs involved in the regulation of RNF169 in PAAD. In this study, 77 lncRNAs were identified as potential upstream regulators of the hsa-miR-324-5p/RNF169 axis. We further found that the lncRNA AL049555.1 was significantly upregulated in PAAD and was associated with poorer overall survival in PAAD patients. Correlation analysis showed that there was a positive correlation between AL049555.1 and RNF169 and a negative correlation between AL049555.1 and hsa-miR-324-5p, indicating that AL049555.1 might be a potential upstream target of the hsa-miR-324-5p/RNF169 axis in PAAD. In summary, the AL049555.1/hsa-miR-324-5p/RNF169 axis was proposed as a potential pathway for the functional regulation of RNF169 in PAAD; however, further experiments are warranted to elucidate the details of the underlying mechanisms.

In the past decade, immunotherapy for cancers has made important breakthroughs (Becht et al., 2016). Studies on the TME for advanced immunotherapy have attracted enormous attention (Wu and Dai, 2017). The TME is a complex organized structure that refers to the external environment of tumour cells and is required for the survival of tumour cells. The TME is composed of tumour cells, fibroblasts, immune cells and other types of cells, as well as tumour stromal cells, extracellular matrix (ECM) and other related factors, comprising approximately 15%–85% of all tumour components in PAAD (Zhang J. et al., 2020; Tan et al., 2020).

Increasing evidence shows that immune infiltration and the tumour microenvironment play an important role in the development and progression of tumours (Liu et al., 2020; Sun et al., 2020). Our results showed that RNF169 was correlated with some tumour-infiltrating lymphocytes in PAAD. Specifically, RNF169 had a substantial positive correlation with some infiltrated immune cells, including B cells, CD8^+^ T cells, macrophages, neutrophils, and dendritic cells, in PAAD, which was further supported by the positive correlation of RNF169 with immune markers. These results suggest that RNF169 may participate in the regulation of tumour immunity in PAAD patients.

The efficacy of immunotherapy depends on infiltrated immune cells and the expression of immune checkpoint molecules in the TME (Zhang et al., 2019; Huang et al., 2020). Thus, we evaluated the correlation between RNF169 and immune checkpoints and found that high expression of RNF169 was associated with CTLA4 or CD274, indicating that targeting RNF169 may be a potential approach to improve the efficacy of immunotherapy for PAAD. Thus, disrupting the function of RNF169 with specific inhibitors or blockers may be a promising strategy for immunotherapy in PAAD patients.

To better understand the role of RNF169 in the progression of PAAD, we utilized LinkedOmics to identify its coexpressed genes, and RSF1, REST, STRN and RIF1 were found to be positively correlated with RNF169. Previous studies have shown that these genes are involved in various biological processes of tumour cells. For example, overexpression of remodelling and spacing Factor 1 (RSF1) has been reported to promote the proliferation and survival of tumour cells (Cai et al., 2021); RE1-silencing transcription factor (REST) was reported to regulate cell apoptosis, the cell cycle, cell proliferation, metastasis, invasion and tumorigenesis (Taghvaei et al., 2021); As a tumour promoter, STRN is upregulated in HCC (Du et al., 2020); and Rap1-interacting Factor 1 (RIF1) expression was found to be significantly correlated with clinical stage and prognosis. Our results showed for the first time that there are significant correlations of RNF169 with the expression of RSF1, REST, STRN and RIF1 in PAAD.

KEGG pathway analysis of the genes coexpressed with RNF169 showed that in PAAD, these genes were mainly involved in the JAK-STAT signaling pathway, TNF signaling pathway, and Toll-like receptor signaling pathway. GO term analysis showed that the RNF169 coexpressed genes in PAAD were mainly enriched in Hippo signaling, positive regulation of cell motility and immune response-regulating signaling pathway, PI3K activity, DNA-binding transcription repressor activity, and RNA polymerase II-specific and Rho GTPase binding. These results indicate the complexity of the functions of RNF169 in tumorigenesis and progression, which are regulated by multiple signaling pathways in PAAD.

The ubiquitination and deubiquitination of proteins plays an important role in tumorigenesis (Li et al., 2015; Lv et al., 2020). Deubiquitylating enzymes (DUBs) have been reported to regulate the ubiquitination process by counteracting the activities of E3 ligases in human cancer (Zhu et al., 2019). As an E3 ubiquitin ligase, RNF169 may rely on ubiquitin to regulate the tumorigenesis of PAAD patients.

Although we collected PAAD data from various public databases to analyse the clinical value of RNF169 in PAAD patients, there are some limitations to this study. First, some results from multiple databases are controversial due to inconsistencies caused by different sample sizes and tumour types. Second, our findings based on bioinformatic analysis need further biological experiments to verify the conclusion; for example, the correlations between RNF169 expression and cytokines, chemokines and chemokine receptors need to be further verified with detailed experiments in the future.

In summary, we utilized multiple open-source databases to evaluate the prognostic value of RNF169 and to dissect the potential mechanisms involved in the development of PAAD. Our results showed that the mRNA level of RNF169 is significantly elevated in PAAD, which is associated with a poor outcome in PAAD patients. RNF169 was found to have a significant correlation with infiltrated immune cells in the TME, indicating a key role of RNF169 in immune cell infiltration in PAAD. The lncRNA AL049555.1 may participate in the regulation of PAAD by RNF169 expression by sponging hsa-miR-324-5p. These results might shed new light on the mechanisms of tumorigenesis and provide new therapeutic targets or diagnostic biomarkers for PAAD.

## Data Availability

The original contributions presented in the study are included in the article/Supplementary Material, further inquiries can be directed to the corresponding authors.
