# Supplementary material for: High expression of RNF169 is associated with poor prognosis in pancreatic adenocarcinoma by regulating tumour immune infiltration
Source: Front Genet. 2023 Jan 5;13:1022626. doi: 10.3389/fgene.2022.1022626 (PMC9849556; doi:10.3389/fgene.2022.1022626)
Supplement: Supplementary file 1 [file Image1.pdf]

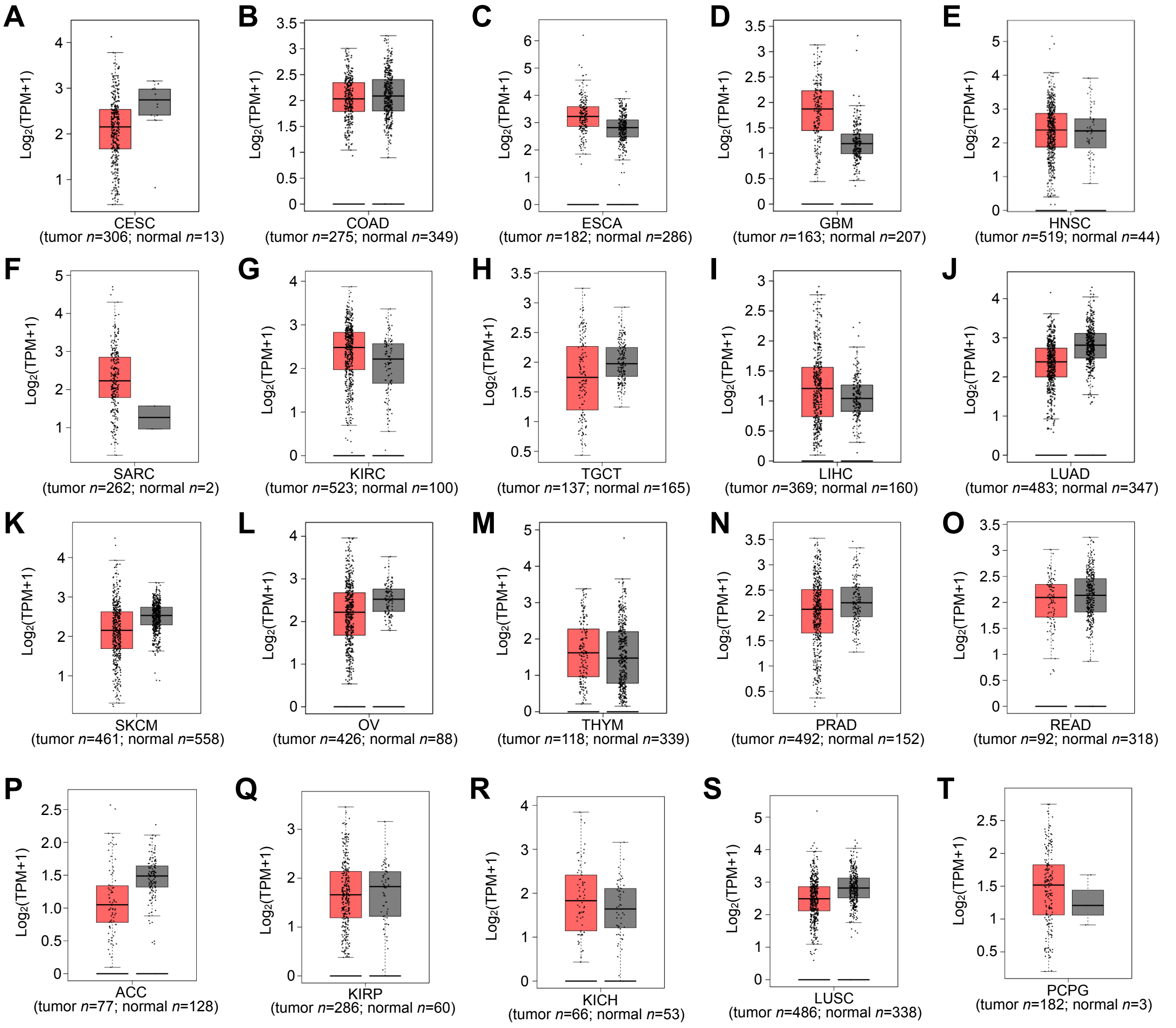

Supplementary Figure 1 Differential expression of RNF169 in cancer and normal tissues in the GEPIA database.

(**A-T**) RNF169 expression in 20 different types of human cancers, including CESC (**A**), COAD (**B**), ESCA (**C**), GBM (**D**), HNSC (**E**), SARC (**F**), KIRC (**G**), TGCT(**H**), LIHC (**I**), LUAD (**J**), SKCM (**K**), OV (**L**), THYM (**M**), PRAD (**N**), READ (**O**), ACC (**P**), KIRP (**Q**), KICH (**R**), LUSC (**S**), and PCPG (**T**), in comparison with adjacent normal tissues.

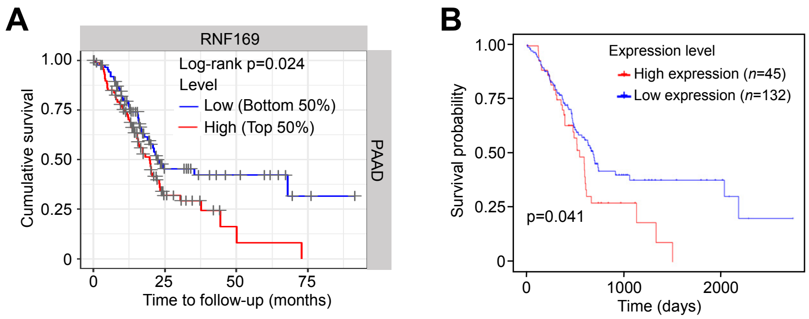

Supplementary Figure 2 The association of RNF169 expression with the survival of PAAD patients.

(**A**) Analysis of cumulative survival of PAAD patients with high or low RNF169 expression using TIMER database. (**B**) The survival probability of PAAD patients with high or low expression of RNF169 analyzed by UALCAN database.

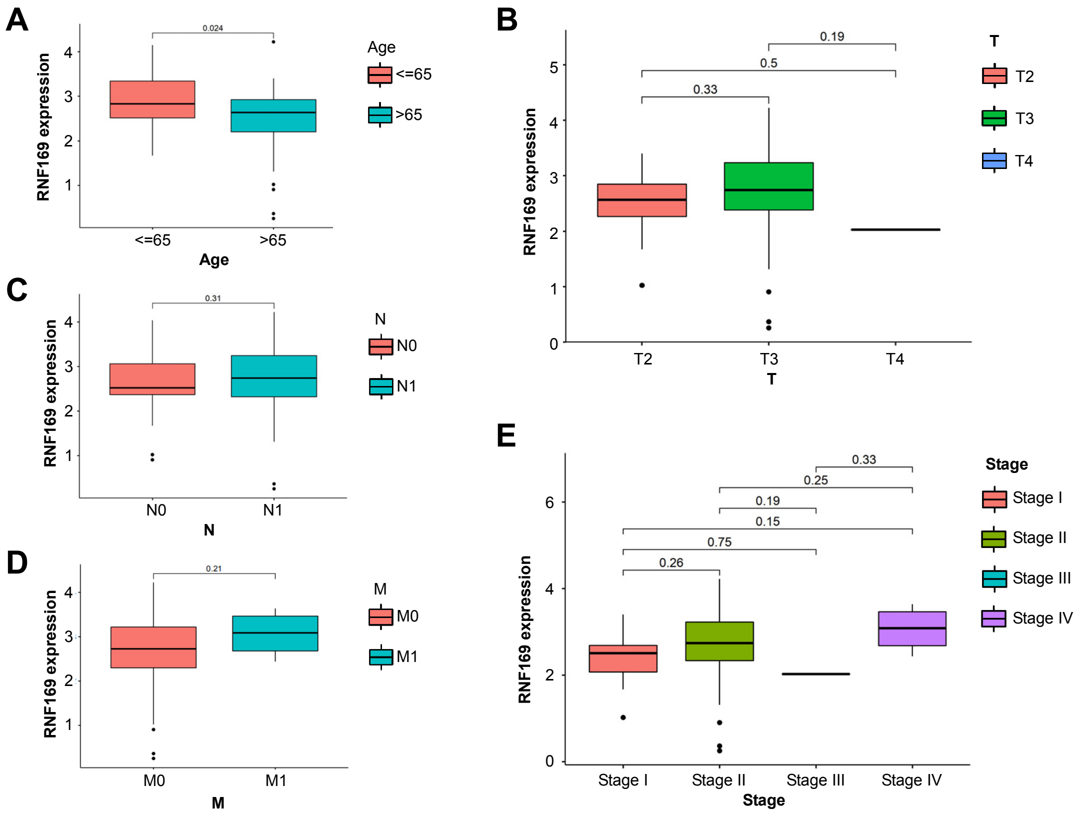

Supplementary Figure 3 Associations between RNF169 expression and clinical traits.
(**A-E**) Different clinical characteristics, including age (**A**), T (**B**), N0-N1 (**C**), M0-M1 (**D**) and stage (**E**) assessed by Wilxcon.

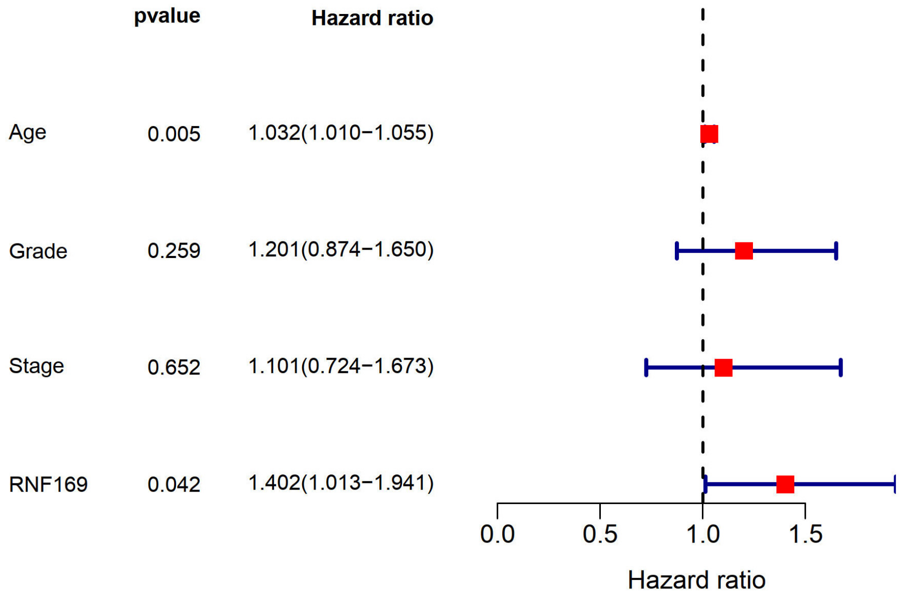

Supplementary Figure 4 Multivariate Cox analysis for the TCGA cohort. MultiCox analysis incorporating age, grade, stage and RNF169 expression shows RNF169 as an independent prognostic factor with a hazard ratio of 1.402 in PAAD patients.

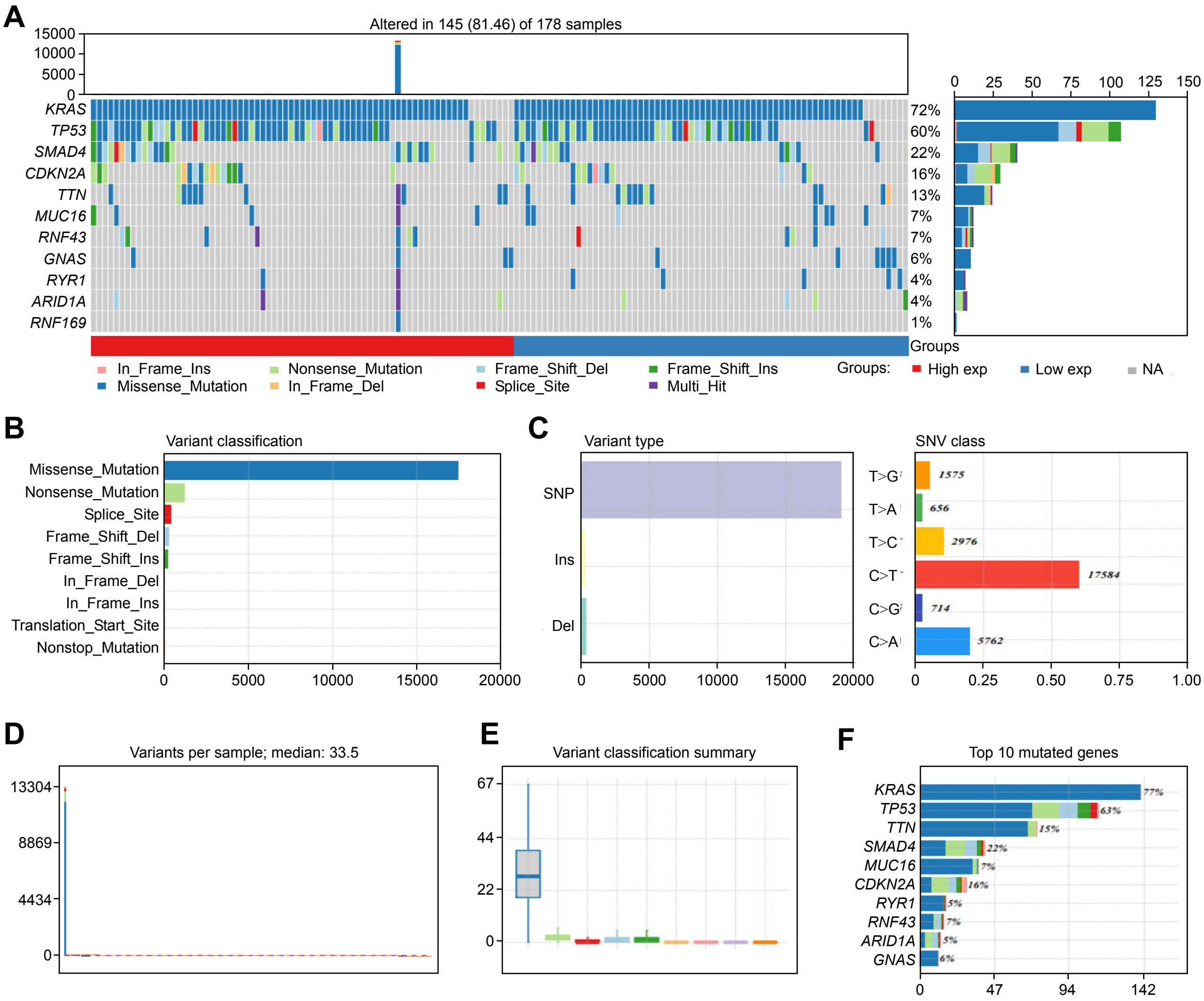

Supplementary Figure 5 Gene mutation information of RNF169 in comparison with frequently mutated genes in PAAD.

(**A**) The mutation types of RNF169 in comparison with the top 10 genes with highly frequent mutations in PAAD. (**B**) The variant classification of RNF169 in PAAD. (**C**) The variant types of RNF169 in PAAD. (**D**) The variants of RNF169 in PAAD samples. (**E**) Statistical information of the variant classification of RNF169 in PAAD. (**F**) The mutation types of the top ten mutated genes in PAAD.

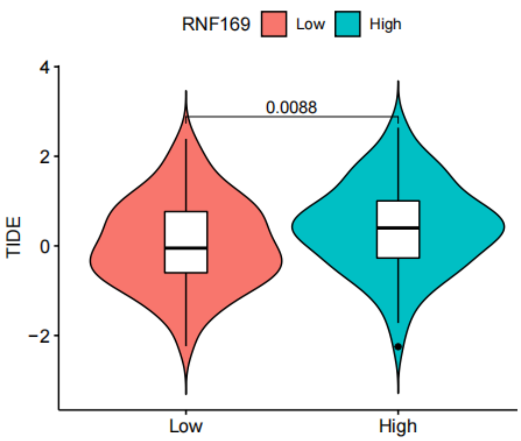

Supplementary Figure 6 Violin plot of Tumor Immune Dysfunction and Exclusion (TIDE) scores in RNF169 high-risk group versus low-risk group in TCGA cohort.

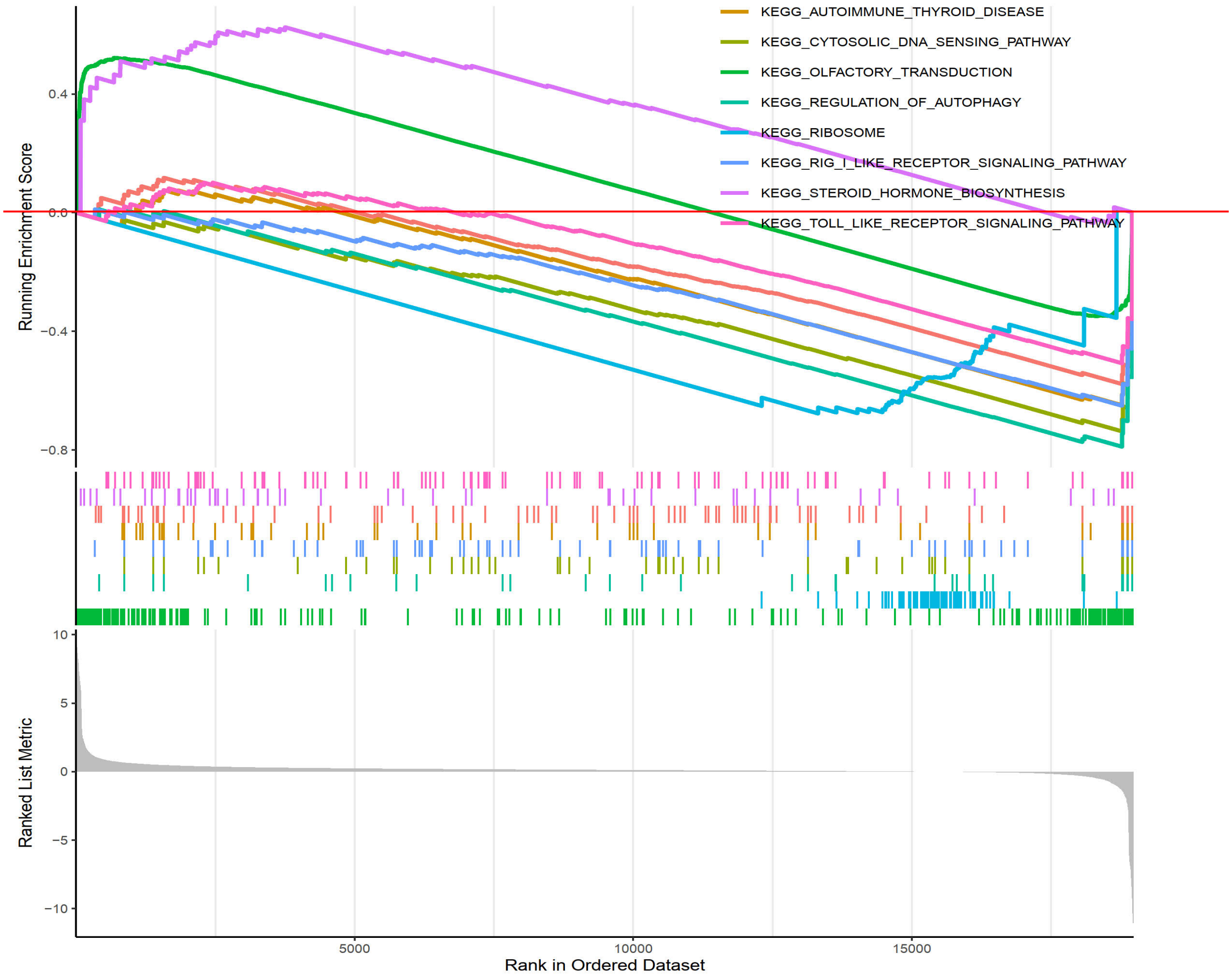

Supplementary Figure 7 GSEA analysis shows signaling pathways related to RNF169 expression.
